# Evaluation of the long-term efficacy of K-Othrine^®^ PolyZone on three surfaces against laboratory reared *Anopheles gambiae* in semi-field conditions

**DOI:** 10.1186/s12936-018-2239-z

**Published:** 2018-02-23

**Authors:** James C. Dunford, Alden S. Estep, Christy M. Waits, Alec G. Richardson, David F. Hoel, Karin Horn, Todd W. Walker, Jessika S. Blersch, Jerry D. Kerce, Robert A. Wirtz

**Affiliations:** 1 Navy and Marine Corps Public Health Center, 620 John Paul Jones Circle, Suite 1100, Portsmouth, VA 23708-2103 USA; 2 Center for Medical, Agricultural, and Veterinary Entomology Detachment, Navy Entomology Center of Excellence, Gainesville, FL 32608 USA; 3 USDA-ARS Center for Medical, Agricultural, and Veterinary Entomology, Gainesville, FL 32608 USA; 4 Lovelace Respiratory Research Institute, Albuquerque, NM 87108 USA; 5 Navy Entomology Center of Excellence, Jacksonville, FL 32212 USA; 6 Navy and Marine Corps Public Health Center Detachment, Centers for Disease Control and Prevention, Atlanta, GA 30341 USA; 7 Bayer CropScience AG, Monheim, Germany; 8 East Baton Rouge Parish Mosquito Abatement and Rodent Control, Baton Rouge, LA 70807 USA; 9 Camp Blanding Joint Training Center, Starke, FL 32091 USA; 10 Centers for Disease Control and Prevention, Atlanta, GA 30341 USA

**Keywords:** K-Othrine PolyZone, Deltamethrin, *Anopheles gambiae*, Experimental huts, Indoor residual spray

## Abstract

**Background:**

In this semi-field study, a new polymer-enhanced deltamethrin formulation, K-Othrine^®^ PolyZone, was compared to a standard deltamethrin product for residual activity against a susceptible strain of laboratory-reared *Anopheles gambiae* using standard WHO cone bioassays.

**Methods:**

Residual insecticide efficacy was recorded after exposure to metal, cement and wood panels maintained in experimental huts in sub-tropical environmental conditions in north central Florida, USA, and panels stored in a climate controlled chamber located at the Centers for Disease Control and Prevention, Georgia, USA.

**Conclusions:**

K-Othrine^®^ PolyZone demonstrated 100% control on metal and cement panels 1 year post application and > 80% control on wood panels up to 6 mo. The new formulation should be considered for use in indoor residual spray programmes requiring long-term control of malaria vectors.

**Electronic supplementary material:**

The online version of this article (10.1186/s12936-018-2239-z) contains supplementary material, which is available to authorized users.

## Background

Indoor residual spraying (IRS) is one of the two most common interventions for control of malaria vectors [1] and is an integral part of the President’s Malaria Initiative (PMI) and Amazon Malaria Initiative to reduce malaria transmission in sub-Saharan Africa and Central and South America. Although an estimated 185 million people are covered under IRS programmes, this represents less than 10% of the people at risk for malaria worldwide. Even with such limited coverage, diligent application of IRS, along with expanding long-lasting insecticidal net (LLIN) distribution programmes, are driving a decrease in worldwide malaria incidence and mortality [1] as well as in focal regions of transmission [2].

Beginning in the late 1940’s, national IRS programmes have been tremendously effective in reducing and in some cases nearly eliminating malaria [1]. When dichloro-diphenyl-trichloroethane (DDT) was used for IRS in the 1950’s, malaria was nearly eradicated from a number of countries in Africa and was successfully eliminated from several islands, many of which still remain malaria-free. Due to concerns over non-target effects [3], contamination and widespread insecticide resistance in mosquitoes [4, 5], other less persistent compounds have been used for IRS since that time. Universal bed net coverage in PMI countries has greatly assisted in the dramatic decrease of malaria deaths since 2000 [6], with IRS playing a more limited role due to cost and logistical constraints. However, several issues have led to failure of IRS programmes and reemergence of malaria burdens. These causes of failure include lack of national government programme support, a shortfall of necessary funding to cover a significant proportion of malaria-endemic countries, and development of insecticide resistance [7, 8]. To bolster these national programmes and ensure the continued decrease in malaria transmission, the World Health Organization (WHO) developed a set of guidelines for IRS in 2013 that recommends best policies and practices for spray programmes. The guidelines also define seven elements of an effective IRS insecticide, among which human safety, efficacy against vectors, and long-term residual effect are noted as critical factors.

The WHO [9] defines satisfactory residual activity as a > 80% mortality rate following exposure to a treated surface, but few insecticides achieve WHO standards beyond 6 months due to a variety of application and environmental variables. The cost effectiveness and residual control of DDT are well documented with efficacy greater than 10 months depending on dosage and substrate [1, 10]; however, few comparable long-lasting alternatives exist. Several studies have demonstrated sustained efficacy (> 70%) of various pyrethroid insecticides on substrates for 6 months or less [11–14], requiring two or more yearly applications depending on regional malaria transmission cycles to provide sufficient control.

Although WHO-approved insecticides include organochlorines, organophosphates, and carbamates, the largest class is the pyrethroids as they tend to have low mammalian toxicity, provide quick knockdown and are relatively inexpensive as compared to the other classes; thus, there tends to be a greater acceptance by homeowners to allow pyrethroids to be applied indoors [11] and support from national malaria control programmes in affected countries. Among the pyrethroids, the type II cyano-pyrethroids have been effectively used for IRS. Lambda-cyhalothrin has been tested in Asia and Africa with good efficacy and residual activity [14, 15]. In comparison to bendiocarb (carbamate) and malathion (organophosphate), use of lambda-cyhalothrin has resulted in better efficacy and in many cases longer residual effect [15]. In both cases, lambda-cyhalothrin has better acceptance than the more noxious carbamates or organophosphates. Deltamethrin, another type II pyrethroid, has also been used in Asia, Iran, and Africa [13, 16, 17]. Many of these studies note significant differences in efficacy and duration on surfaces of varied porosity and with different formulations.

The *Anopheles gambiae* complex has historically been considered the most important vectors of malaria in Africa [18] and the target for control using IRS. In this semi-field study, a comparison was made between a new formulation of K-Othrine^®^ PolyZone, a polymer enhanced suspension concentrate aqueous formulation containing 62.5 g deltamethrin per liter, to K-Othrine^®^ WG250 and control spray for residual activity against a pyrethroid susceptible strain of laboratory-reared *Anopheles gambiae*. Residual efficacy on plywood, metal, and cement panels maintained in experimental huts was evaluated under sub-tropical environmental conditions in north central Florida, USA. Panels from experimental huts positioned in full sun, partial shade, and fully shaded locations were compared to panels maintained under climate controlled conditions in an environmental chamber located at the Centers for Disease Control and Prevention (CDC) to determine longevity of both insecticides on three surfaces as affected by climatological variation. Detachable panels were tested in CDC laboratories using standard WHO cone bioassays and then returned to the original experimental huts to continue aging. This study extends previous laboratory work with K-Othrine^®^ products conducted by Vatandoost et al. [12] into a semi-field evaluation.

## Methods

This study was designed to examine residual efficacy of IRS subject to four factors; treatment, surface type, exposure, and time. These factors are described in more detail below. Each combination of factors was replicated three times. All spray mixes were prepared and sprayed under the supervision of DoD-certified pest applicators and followed local environmental regulations.

Abandoned military latrines (see Additional file 1) (3 m × 2.5 m × 3 m) located at the Florida National Guard, Camp Blanding Joint Training Center (CBJTC) in north central Florida, USA (see Additional file 2) were used as experimental huts to hold panels treated with water, traditionally formulated deltamethrin (K-Othrine^®^ WG 250, Bayer), and K-Othrine^®^ PolyZone (a.i. 62.5 g/L deltamethrin; Bayer, WHOPES recommendation achieved September 2013) to determine residual efficacy on three different surfaces exposed to natural environmental conditions. Panels of plywood (true wood, non-treated) and galvanized steel were cut to the size of 30.5 by 30.5 cm^2^. The cement panels were made using the same plywood base with a 2.5 cm lathe base and sand concrete mix (Sakrete, Oldcastle Architectural, FL, USA). Cement panels were allowed to cure for 2 weeks prior to treatment. A 4 mm hole was drilled in the corner of each panel (before treatment) to facilitate hanging. Each surface type and treatment was replicated in three separate huts where they were subjected to ambient temperature (ranging between − 2 to 40 °C) (see Additional file 3) and humidity (26–96%) variations during the experimental time period of 1 year. An additional set of panels made of the same materials were treated at CBJTC and maintained in an insectary environmental chamber located at the CDC, Entomology Branch, Georgia, USA. Environmental conditions in the chamber were maintained at 28 °C, ~ 77% RH throughout the study.

To maintain environmental integrity and adhere to CBJTC requirements, 6 mill plastic was placed on the ground and all materials were laid on the plastic for spray application. Traditionally, the materials would be vertically oriented but in this study our goals were to assess the efficacy of the same active ingredient in different formulations and to assess the efficacy of these formulations on different surfaces. Thus, all panels were treated horizontally so that run-off from the less porous metal surface did not reduce the actual active ingredient concentration versus the other surface types. To further reduce runoff from the surfaces, all panels were allowed to air-dry for 1 h after treatment. Treatment blocks were spaced roughly 30–60 m apart, perpendicular to the prevailing wind to ensure no contamination between treatments. Panels were treated with one of three treatments: water as control (CTL), K-Othrine^®^ WG 250 (WG 250), Bayer and K-Othrine^®^ PolyZone (Deltamethrin SC 62.5, Bayer (PolyZone)). Tap water from an outdoor faucet at the Navy Entomology Center of Excellence (NECE) was used. The three surface types were randomly mixed and sprayed together for each treatment to reduce variability. Each product was applied at 20 mg/m^2^ (label rate) to 8.92 square meters of panel surface using a hand can (Hudson X-Pert, Chicago, IL, USA) with a flat fan nozzle (TeeJet 8002, Spray Systems Co., Bessemer, AL, USA) pumped to maximum pressure. Each treatment was applied with a different hand can using the same nozzle type and psi. The spraying was conducted at a standard rhythm recommended by WHO [1].

Following treatment and drying, the panels were hung in shelters with a screw through the predrilled hole, with the exception of the panels treated for the chamber at the CDC. The huts were placed in locations with exposure to different amounts of sunlight/tree shading (see Additional file 2): 3 huts received full sun (#’s 1, 2, 3), 3 huts partial sun (#’s 4, 5, 6), and 3 huts full shade (#’s 7, 8, 9). Each hut received panels from the three spray treatments of one surface type (wood, cement, metal). Hut #2 received two surface types due to the presence of beneficial wasps in hut #1 which required it to be abandoned. All huts were equipped with a HOBO U12-012 datalogger (Onset Computer Corp, MA, USA) to record temperature and relative humidity. No weather data was collected for the period from 20 February through 1 April 2015, when no monthly testing was performed. The field panels were packaged individually in two-gallon Ziploc bags and transported to the CDC for testing. Personnel conducting panel collections from experimental huts used nitrile gloves while handling panels on the edges and corners.

Panels were evaluated for residual efficacy using standard WHO cone bioassays [9] against *An. gambiae* (G3 pyrethroid susceptible strain) maintained in colony at the CDC. Panels were positioned flat on a surface during bioassays (see Additional file 4) because they were treated with insecticide in a flat position in the field. The G3 strain was obtained through the MR4 as part of the BEI Resources Repository, NIAID, NIH: *Anopheles gambiae* G3, MRA-112, deposited by MQ Benedict. *Anopheles gambiae* females (approximately 10/cone, 5 cones/panel were exposed in five WHO cones to each treated panel surface for 30 min (see Additional file 4). Knockdown counts were taken at 30 min; after which mosquitoes were transferred to screened holding containers (Neptune Paper Co, NJ, USA), provided 10% sugar solution, and held overnight at 28 °C, ~ 77% RH. Mortality counts were taken again at 24-h post exposure.

Statistical analysis of 24 h mortality and 30 min knockdown data was performed using a general linear model as implemented by R version 3.4.3 as has been described previously [19, 20], (see Additional files 5, 6). To maintain the binomial requirement for this analysis, the response variable (knockdown or mortality) was calculated by the programme from the raw counts knocked down or dead out of the total possible rather than starting with proportional response data. The dataset for analysis consisted of 30-min and 24-h mosquito count data with 4 factors: (1) 3 treatments: CTL, WG 250, PolyZone; (2) 3 surfaces: wood, cement, metal; (3) 4 locations: full sun, partial sun, full shade, CDC chamber; (4) 11 time points: 0.25, 1, 2, 3, 4, 5, 6, 7, 8, 9, and 12 mo. Initial *glm* analysis included a term for the interaction between surface and treatment as well as the main factors (see Additional files 5, 6). When possible, the initial model was stepped down by removing non-significant factors and rerunning the analysis as noted in the additional files. Post hoc means comparison was performed using the *glht* function in the *multcomp* package. Raw data is included in Additional files 5 and 6. Tukey comparisons were made to determine pair-wise significant effects of treatment and surface type when possible [21].

## Results

WHO cone bioassay testing was utilized with a susceptible strain (G3) of *An. gambiae* to examine a variety of factors; environmental exposure, surface type, and treatment. Panel sampling and testing were performed 11 times over a 1 year period to determine the effective period of these treatments. Specific surface composition, pesticide doses, and collection/testing time points are described in the Methods section. Initial 30-min knockdown and 24-h mortality were measured.

WHO cone bioassay testing of Polyzone and WG-250 treated panels indicated significant effects in comparison to the control panels maintained in the CDC environmental control chambers. Modeling the 30 min knockdown indicated two significant factors. Panels maintained in the controlled environmental chamber at CDC were more effective than panels in experimental huts exposed to full sun, partial sun or fully shaded but these three field exposures were not different from one another. A significant decline in knockdown was observed as panels aged; however there was not a significant contribution to knockdown from the interaction between surface and treatment.

**Fig. 1:**
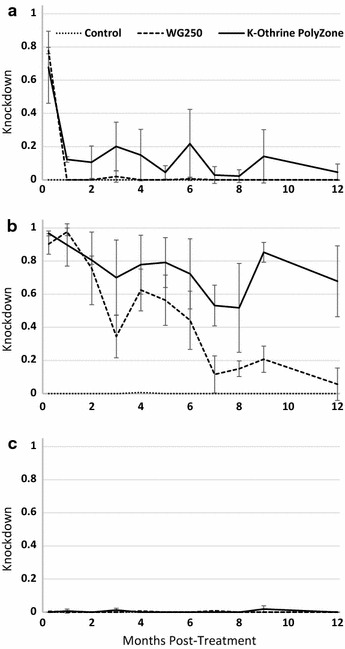
*Anopheles gambiae* knockdown at 30 min on treated panels. **a** metal; **b** cement; **c** wood. Control = water treated control panels aged at Camp Blanding Joint Training Center field site. WG 250 = panels treated with K-Othrine^®^ WG250 aged at Camp Blanding Joint Training Center field site. K-Othrine^®^ PolyZone = panels treated with deltamethrin suspension concentrate (K-Othrine^®^ PolyZone) aged at Camp Blanding Joint Training Center field site

The substrate type clearly affects the efficacy of the IRS products. On metal panels, the PolyZone and WG-250 treated panels produced more 30 min knockdown than the control panels for the first 9 months of testing (Fig. 1a). Even 1 year after treatment, the PolyZone treated metal panels still produced about 80% knockdown. While the knockdown of the WG-250 treated metal panels was similar during the first 5 months, by the sixth month it was less than the PolyZone treated metal panels. It continued to fade through months 7–9 and, while still higher than the control panels, was less than the K-Othrine PolyZone. By the 1 year point, the knockdown of WG-250 on metal was not higher than the control panels. The cement panels treated with the two deltamethrin treatments did have knockdown at the first testing a week after application but was not significantly greater than the control by the 1 month timepoint (Fig. 1b). The results do show a low level of knockdown is present on the PolyZone treated cement panels. Neither the PolyZone or WG-250 formulations treatment produced knockdown above 10% at any time point on the wood panels (Fig. 1c).

Twenty-four hour mortality also showed significant effects. Panels stored in the CDC environmental chamber were more effective than panels subject to natural temperature and humidity variation regardless of exposure condition. There were also significant effects due to panel treatment, panel material, and the interaction between the two factors with mortality that in most cases faded as the panels aged.

**Fig. 2:**
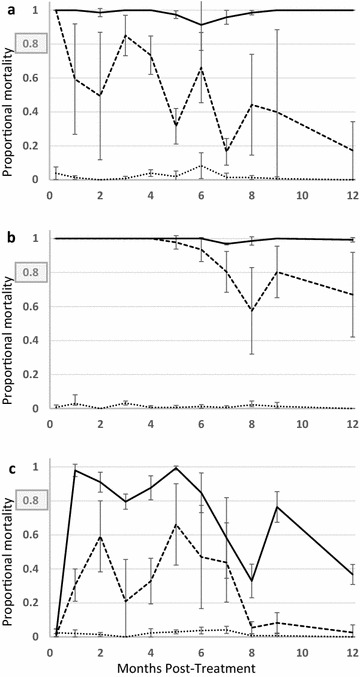
*Anopheles gambiae* mortality at 24 h on treated panels. **a** metal; **b** cement; **c** wood. The boxed 0.8 on the vertical scale represents the 80% mortality level considered effective under WHO guidance. Control = water treated control panels aged at Camp Blanding Joint Training Center field site. WG 250 = panels treated with K-Othrine^®^ WG250 aged at Camp Blanding Joint Training Center field site. K-Othrine^®^ PolyZone = panels treated with deltamethrin suspension concentrate (K-Othrine^®^ PolyZone) aged at Camp Blanding Joint Training Center field site

As with 30 min knockdown variation in efficacy on the different substrates was observed. The metal panels treated with K-Othrine PolyZone produced greater than 98% 24 h mortality at every test period during the year (Fig. 2a). The WG-250 matched this through the first 5 months. After this, the WG-250 produced a trend of lower mortality (60–80%) but was still higher than the control panels. PolyZone treatment also produced high mortality on the cement panels during the entire testing period (Fig. 2b). Even though the WG-250 panels contained the same dose of active ingredient, the 24 h mortality was less than the PolyZone treated cement panels and often below the WHO 80% efficacy threshold. On the wood surfaces, the first testing period at 1 week after application did not produce any mortality regardless of the treatment (Fig. 2c). However, by 1 month the K-Othrine Polyzone treatment was effective and caused mortality of nearly all of the test organisms.

## Discussion

Several things should be considered when initiating and conducting IRS operations. Optimum effectiveness of IRS efforts is achieved by using the correct formulation for the type of surface being treated. For instance, wettable powders (WP) and water dispersible granules (WG) are best suited for very porous surfaces such as mud walls, while suspension concentrates (SC) or emulsifiable concentrates (EC) are more effective on finished cement, finished wood or lumber, or painted surfaces, especially those where oil-based paints have been applied [1]. This study demonstrated that K-Othrine^®^ PolyZone (SC) outperformed a conventional deltamethrin product, K-Othrine^®^ WG-250, especially with respect to residual mortality on metal and cement surfaces. Although wood panels demonstrated lower overall mortality and residual, K-Othrine^®^ PolyZone provided > 80% control in field-aged panels for up to 6 months, nearly two times greater than control provided by the other deltamethrin product we tested. The interaction on wood panels is intriguing because an efficacy lag with both deltamethrin formulations was observed at the initial 1 week testing period. While there may be no specific explanation for this observation, efficacy was strong at the 1 month time point begging the question of whether this initial lack of efficacy was an issue of availability before the formulation began to weather. It is not expect that it was a panel issue as each treatment was replicated on three separate panels and the efficacy of each individual panel was low.

Along with chosen formulation, the frequency of spraying cycles should be considered and will depend on the malaria transmission patterns in a given region. According to the WHO, spray rounds should ideally be completed in less than 2 months and just before the start of the peak transmission season in holoendemic areas (generally, just prior to the beginning of the rainy season). In endemic areas with perennial transmission, two rounds of spraying in 6 months cycles may be recommended to ensure that there is adequate year-round coverage with residual insecticides. If the transmission pattern exhibits bimodal peaks, spraying rounds should target the peaks, beginning just before the first peak; hence, one annual spray with a long-lasting insecticide would hopefully survive through the second peak season and thus result in significant insecticide and operational cost savings. In areas with one seasonal transmission peak, one spray round, in yearly cycles before the period of transmission, should be enough to have an impact on malaria transmission. However, DDT and new capsule suspension (CS) insecticide formulations have been shown, in some areas, to last more than 10 months [1]. Results from this study suggest that use of K-Othrine^®^ PolyZone provides sufficient control for 1 year on the metal and cement surfaces evaluated.

Insecticides with longer residual attributes should also be considered for outdoor surface efficacy evaluations, which may be a necessary intervention given changes in resting and feeding behaviors observed in some malaria vectors. *Anopheles* species that were historically endophilic have in some locations shifted to outdoor resting/feeding preferences [22, 23]; however, to date there is no commonly used intervention in highly endemic regions such as Africa that specifically targets outdoor biting mosquitoes [24]. The residual activity demonstrated in this study indicates that alternatives to long-lasting DDT are feasible through modern formulation technology. This new formulation of deltamethrin extends the residual efficacy on multiple surfaces, which may require fewer applications, reducing spraying costs, and minimizing disruption to families in highly endemic regions. Further work with other malaria vector species, different housing surfaces, and natural populations under field conditions is warranted to further demonstrate the effectiveness of K-Othrine^®^ PolyZone in a local context before using it in large-scale IRS operations.

## Conclusions

K-Othrine^®^ PolyZone demonstrated 100% control of susceptible *An*. *gambiae* on metal and cement panels 1 year post application and > 80% control on wood panels for up to 6 months. The new formulation should be considered for use in indoor residual spray programmes requiring long-term control of malaria vectors. Further work with other species, different surfaces, and natural populations under field conditions is warranted to further demonstrate the effectiveness of K-Othrine^®^ PolyZone in a local context before using it in large-scale IRS operations.

## Additional files

**Additional file 1.** Representative experimental hut located at Camp Blanding Joint Training Center, Florida, USA.

**Additional file 2.** Map showing locations of ten experimental huts at Camp Blanding Joint Training Center, Florida, USA.

**Additional file 3.** Graphs showing temperature variations in experimental huts located in full sun, partial sun, and full shade from 20 May 2014 to 20 May 2015. No weather data was collected for the period from 20FEB2015–01APR2015 when no monthly testing was performed. Green = MAX Blue = AVG; Red = MIN.

**Additional file 4.** WHO Cone Bioassays on insecticide treated cement panels.

**Additional file 5.** Raw data statistical analysis of 24 h mortality.

**Additional file 6.** Raw data statistical analysis of 30 min knockdown.
