# Supplementary figures and images for: Evaluation of the long-term efficacy of K-Othrine® PolyZone on three surfaces against laboratory reared Anopheles gambiae in semi-field conditions
Source: Malar J. 2018 Feb 23;17:94. doi: 10.1186/s12936-018-2239-z (PMC5824574; doi:10.1186/s12936-018-2239-z)

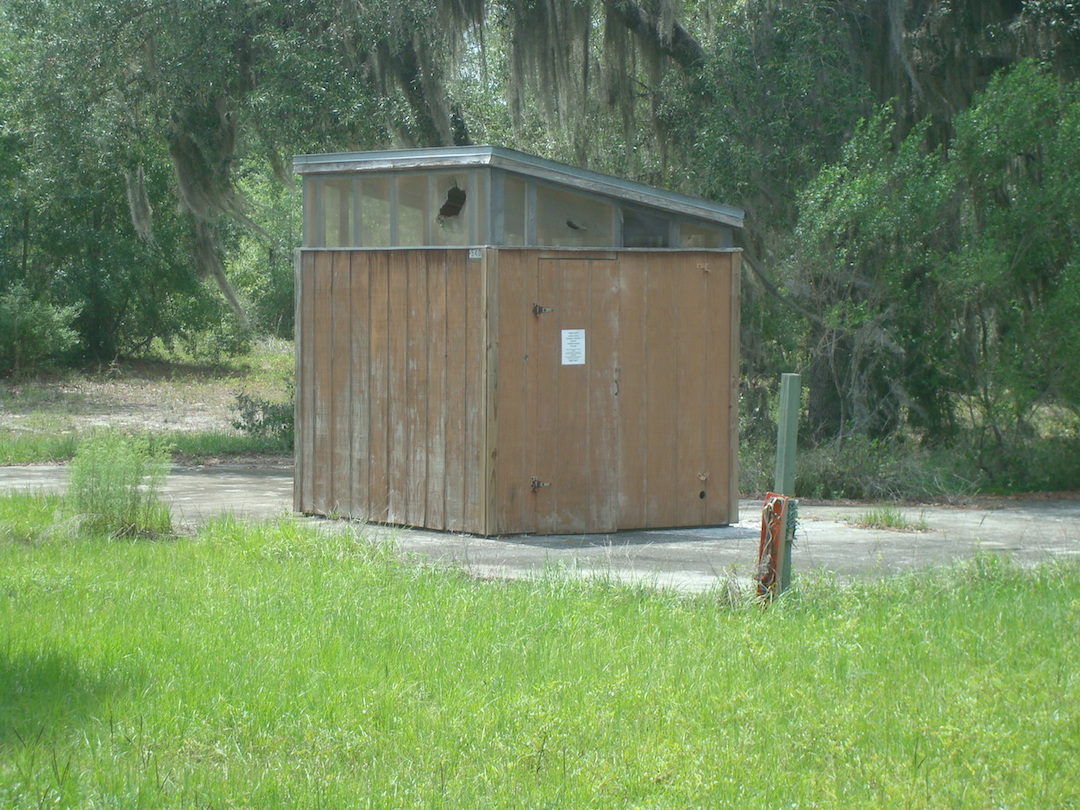

Supplement: Supplementary file 1 — Additional file 1. Representative experimental hut located at Camp Blanding Joint Training Center, Florida, USA. [file 12936_2018_2239_MOESM1_ESM.jpg]

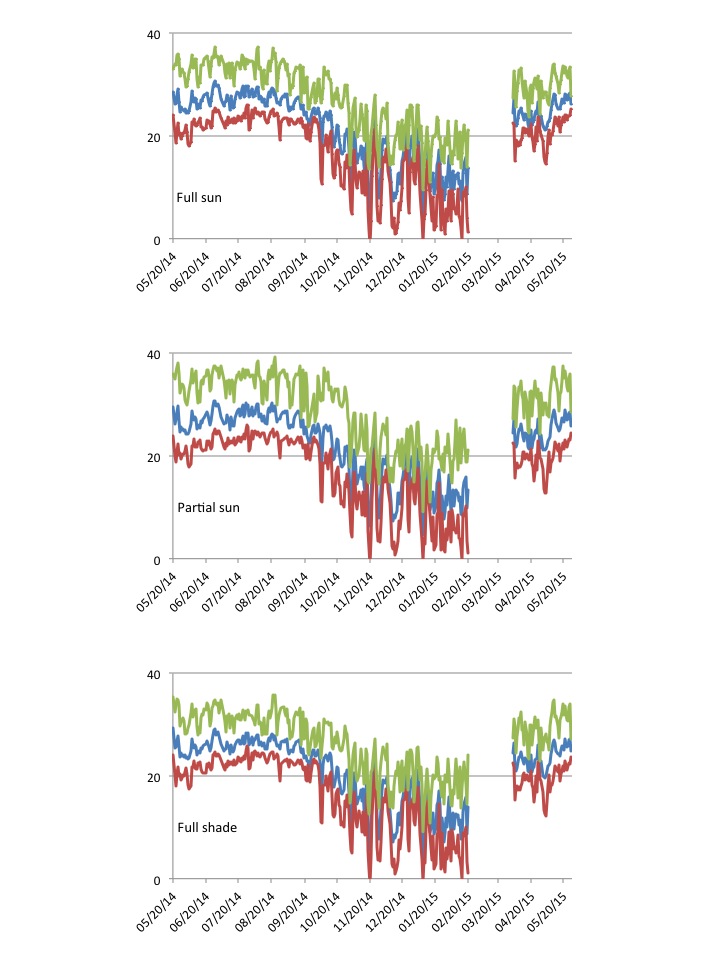

Supplement: Supplementary file 3 — Additional file 3. Graphs showing temperature variations in experimental huts located in full sun, partial sun, and full shade from 20 May 2014 to 20 May 2015. No weather data was collected for the period from 20FEB2015–01APR2015 when no monthly testing was performed. Green = MAX Blue = AVG; Red = MIN. [file 12936_2018_2239_MOESM3_ESM.jpg]

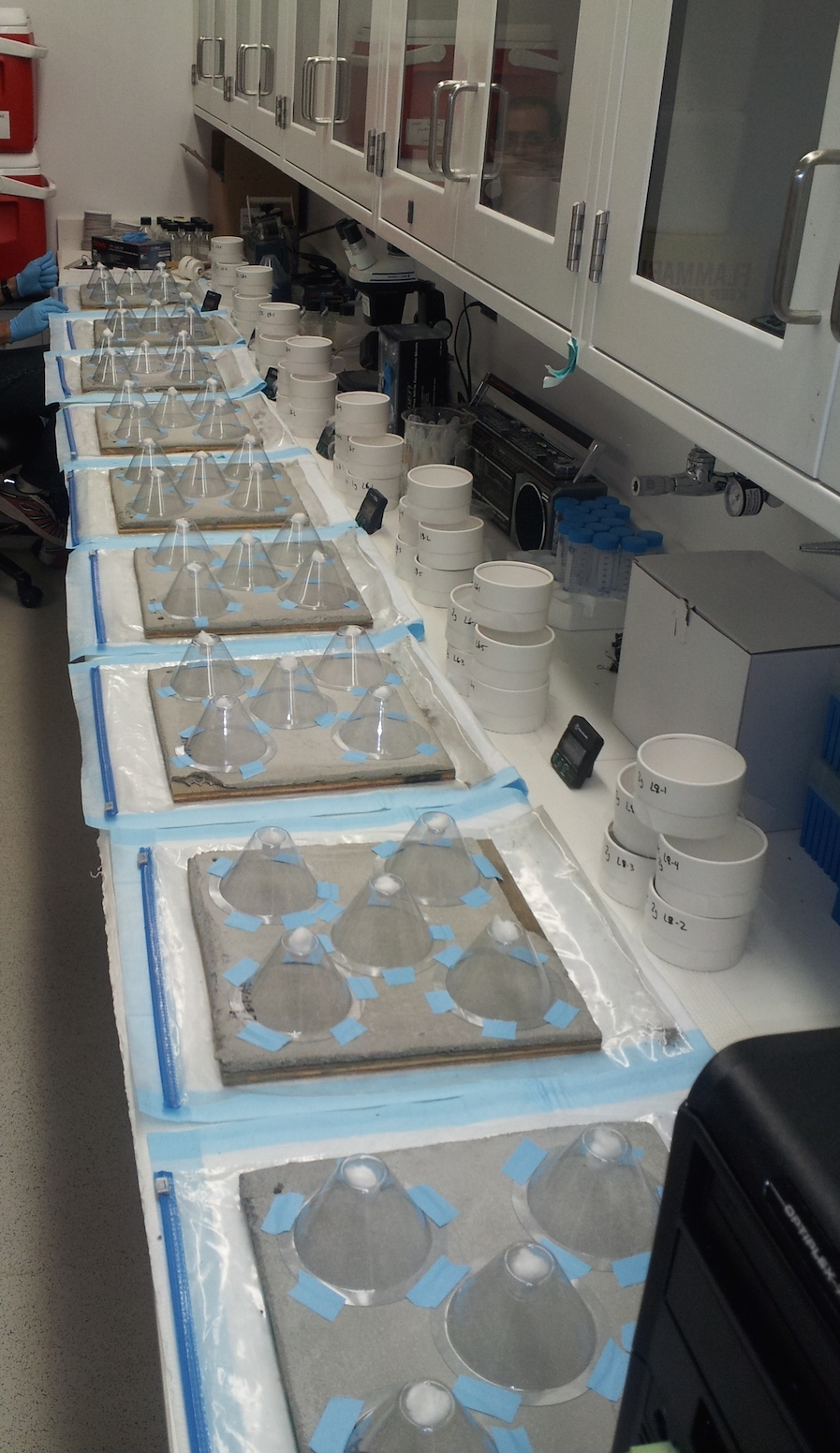

Supplement: Supplementary file 4 — Additional file 4. WHO Cone Bioassays on insecticide treated cement panels. [file 12936_2018_2239_MOESM4_ESM.jpg]
