# Supplementary material for: Evaluation of the long-term efficacy of K-Othrine® PolyZone on three surfaces against laboratory reared Anopheles gambiae in semi-field conditions
Source: Malar J. 2018 Feb 23;17:94. doi: 10.1186/s12936-018-2239-z (PMC5824574; doi:10.1186/s12936-018-2239-z)

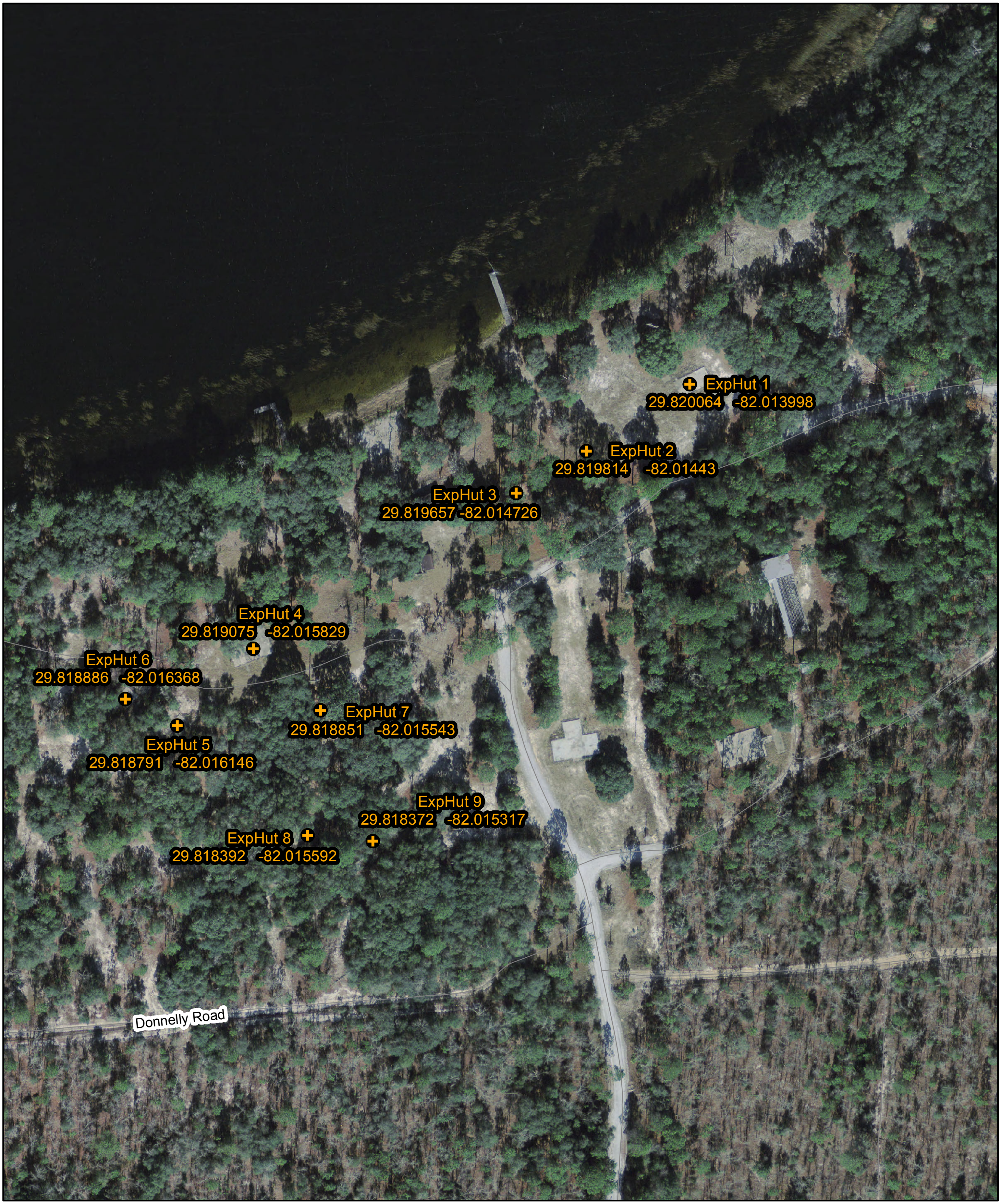

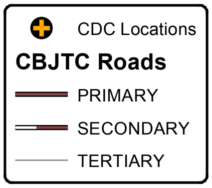

Camp Blanding Joint Training Center
Starke, Florida 32091
Locations of Experimental Huts used for Panel Aging, R2 Training Area

1:2,000

0 75 150 300 450 600 Feet

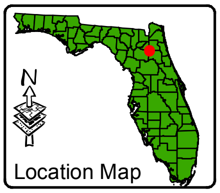

Supplement: Supplementary file 2 — Additional file 2. Map showing locations of ten experimental huts at Camp Blanding Joint Training Center, Florida, USA. [file 12936_2018_2239_MOESM2_ESM.pdf]
