# Supplementary material for: Evaluation of the long-term efficacy of K-Othrine® PolyZone on three surfaces against laboratory reared Anopheles gambiae in semi-field conditions
Source: Malar J. 2018 Feb 23;17:94. doi: 10.1186/s12936-018-2239-z (PMC5824574; doi:10.1186/s12936-018-2239-z)
Supplement: Supplementary file 6 — Additional file 6. Raw data statistical analysis of 30 min knockdown. [file 12936_2018_2239_MOESM6_ESM.pdf]

```
R version 3.4.3 (2017-11-30) -- "Kite-Eating Tree"
Copyright (C) 2017 The R Foundation for Statistical Computing
Platform: x86_64-w64-mingw32/x64 (64-bit)

R is free software and comes with ABSOLUTELY NO WARRANTY.
You are welcome to redistribute it under certain conditions.
Type 'license()' or 'licence()' for distribution details.

  Natural language support but running in an English locale

R is a collaborative project with many contributors.
Type 'contributors()' for more information and
'citation()' on how to cite R or R packages in publications.

Type 'demo()' for some demos, 'help()' for on-line help, or
'help.start()' for an HTML browser interface to help.
Type 'q()' to quit R.

> mydata = read.table("D:/Office_C_drive/Documents/Manuscripts/K-Orthine IRS/latest/All_data.csv"
, header=TRUE, fill=TRUE, sep=";")
> mydata$Time=factor(mydata$Time)
> summary(mydata)
       X                 Exposure       Site     Treatment   Surface   
 Min.   :  1.00   Chamber    :99   CDC    : 99   CTL:132   Cement:132  
 1st Qu.: 99.75   Full_Sun   :99   L1     : 33   DLT:132   Metal :132  
 Median :198.50   Partial_Sun:99   L2     : 33   DSC:132   Wood  :132  
 Mean   :198.50   Shade      :99   L3     : 33                         
 3rd Qu.:297.25                    L4     : 33                         
 Max.   :396.00                    L5     : 33                         
                                   (Other):132                         
      Time          KD30            Dead24          Total     
 0.25   : 36   Min.   : 0.000   Min.   : 0.00   Min.   :24.0  
 1      : 36   1st Qu.: 0.000   1st Qu.: 1.00   1st Qu.:47.0  
 2      : 36   Median : 0.000   Median :22.00   Median :50.0  
 3      : 36   Mean   : 9.288   Mean   :25.09   Mean   :50.6  
 4      : 36   3rd Qu.: 9.000   3rd Qu.:47.00   3rd Qu.:53.0  
 5      : 36   Max.   :80.000   Max.   :84.00   Max.   :84.0  
 (Other):180                                                  
> mydata
      X    Exposure Site Treatment Surface Time KD30 Dead24 Total
1     1    Full_Sun   L1       CTL    Wood 0.25    0      1    24
2     2 Partial_Sun   L4       CTL    Wood 0.25    0      0    79
3     3       Shade   L7       CTL    Wood 0.25    0      2    65
4     4     Chamber  CDC       CTL    Wood 0.25    0      1    61
5     5    Full_Sun   L1       DLT    Wood 0.25    0      1    69
6     6 Partial_Sun   L4       DLT    Wood 0.25    0      0    58
7     7       Shade   L7       DLT    Wood 0.25    1      1    63
8     8     Chamber  CDC       DLT    Wood 0.25    0      1    60
9     9    Full_Sun   L1       DSC    Wood 0.25    0      0    70
10   10 Partial_Sun   L4       DSC    Wood 0.25    0      0    76
11   11       Shade   L7       DSC    Wood 0.25    0      4    77
12   12     Chamber  CDC       DSC    Wood 0.25    1      1    63
13   13    Full_Sun   L2       CTL  Cement 0.25    0      2    46
14   14 Partial_Sun   L5       CTL  Cement 0.25    0      0    59
15   15       Shade   L8       CTL  Cement 0.25    0      4    55
16   16     Chamber  CDC       CTL  Cement 0.25    0      2    53
17   17    Full_Sun   L2       DLT  Cement 0.25   52     68    68
18   18 Partial_Sun   L5       DLT  Cement 0.25   47     61    61
19   19       Shade   L8       DLT  Cement 0.25   40     50    50
20   20     Chamber  CDC       DLT  Cement 0.25   25     58    58
21   21    Full_Sun   L2       DSC  Cement 0.25   57     62    62
22   22 Partial_Sun   L5       DSC  Cement 0.25   27     44    44
23   23       Shade   L8       DSC  Cement 0.25   26     52    52
24   24     Chamber  CDC       DSC  Cement 0.25   42     56    56
25   25    Full_Sun   L3       CTL   Metal 0.25    0      0    50
26   26 Partial_Sun   L6       CTL   Metal 0.25    0      0    61
27   27       Shade   L9       CTL   Metal 0.25    0      1    43
28   28     Chamber  CDC       CTL   Metal 0.25    0      0    41
29   29    Full_Sun   L3       DLT   Metal 0.25   35     42    42
```

30	30	Partial_Sun	L6	DLT	Metal	0.25	61	66	66
31	31	Shade	L9	DLT	Metal	0.25	80	84	84
32	32	Chamber	CDC	DLT	Metal	0.25	45	48	48
33	33	Full_Sun	L3	DSC	Metal	0.25	55	56	56
34	34	Partial_Sun	L6	DSC	Metal	0.25	59	61	61
35	35	Shade	L9	DSC	Metal	0.25	41	43	43
36	36	Chamber	CDC	DSC	Metal	0.25	39	40	40
37	37	Full_Sun	L1	CTL	Wood	1	0	2	47
38	38	Partial_Sun	L4	CTL	Wood	1	0	0	53
39	39	Shade	L7	CTL	Wood	1	0	1	54
40	40	Chamber	CDC	CTL	Wood	1	0	0	51
41	41	Full_Sun	L1	DLT	Wood	1	0	20	49
42	42	Partial_Sun	L4	DLT	Wood	1	0	13	46
43	43	Shade	L7	DLT	Wood	1	0	10	45
44	44	Chamber	CDC	DLT	Wood	1	0	3	47
45	45	Full_Sun	L1	DSC	Wood	1	1	47	47
46	46	Partial_Sun	L4	DSC	Wood	1	0	50	50
47	47	Shade	L7	DSC	Wood	1	0	45	48
48	48	Chamber	CDC	DSC	Wood	1	0	36	40
49	49	Full_Sun	L2	CTL	Cement	1	0	1	56
50	50	Partial_Sun	L5	CTL	Cement	1	0	0	50
51	51	Shade	L8	CTL	Cement	1	0	1	49
52	52	Chamber	CDC	CTL	Cement	1	0	0	59
53	53	Full_Sun	L2	DLT	Cement	1	0	12	55
54	54	Partial_Sun	L5	DLT	Cement	1	0	42	54
55	55	Shade	L8	DLT	Cement	1	0	44	56
56	56	Chamber	CDC	DLT	Cement	1	2	53	53
57	57	Full_Sun	L2	DSC	Cement	1	6	57	57
58	58	Partial_Sun	L5	DSC	Cement	1	7	56	56
59	59	Shade	L8	DSC	Cement	1	7	51	51
60	60	Chamber	CDC	DSC	Cement	1	5	53	53
61	61	Full_Sun	L3	CTL	Metal	1	0	0	51
62	62	Partial_Sun	L6	CTL	Metal	1	0	4	45
63	63	Shade	L9	CTL	Metal	1	0	0	42
64	64	Chamber	CDC	CTL	Metal	1	0	0	45
65	65	Full_Sun	L3	DLT	Metal	1	62	65	65
66	66	Partial_Sun	L6	DLT	Metal	1	46	46	46
67	67	Shade	L9	DLT	Metal	1	41	42	42
68	68	Chamber	CDC	DLT	Metal	1	49	49	49
69	69	Full_Sun	L3	DSC	Metal	1	50	52	52
70	70	Partial_Sun	L6	DSC	Metal	1	49	50	50
71	71	Shade	L9	DSC	Metal	1	39	52	52
72	72	Chamber	CDC	DSC	Metal	1	48	48	48
73	73	Full_Sun	L1	CTL	Wood	2	0	1	49
74	74	Partial_Sun	L4	CTL	Wood	2	0	1	46
75	75	Shade	L7	CTL	Wood	2	0	0	49
76	76	Chamber	CDC	CTL	Wood	2	0	1	49
77	77	Full_Sun	L1	DLT	Wood	2	0	35	48
78	78	Partial_Sun	L4	DLT	Wood	2	0	34	49
79	79	Shade	L7	DLT	Wood	2	0	19	54
80	80	Chamber	CDC	DLT	Wood	2	0	7	49
81	81	Full_Sun	L1	DSC	Wood	2	0	43	44
82	82	Partial_Sun	L4	DSC	Wood	2	0	48	55
83	83	Shade	L7	DSC	Wood	2	0	44	50
84	84	Chamber	CDC	DSC	Wood	2	0	44	50
85	85	Full_Sun	L2	CTL	Cement	2	0	0	46
86	86	Partial_Sun	L5	CTL	Cement	2	0	0	52
87	87	Shade	L8	CTL	Cement	2	0	0	55
88	88	Chamber	CDC	CTL	Cement	2	0	2	44
89	89	Full_Sun	L2	DLT	Cement	2	0	26	42
90	90	Partial_Sun	L5	DLT	Cement	2	0	38	48
91	91	Shade	L8	DLT	Cement	2	0	3	42
92	92	Chamber	CDC	DLT	Cement	2	0	24	48
93	93	Full_Sun	L2	DSC	Cement	2	4	46	48
94	94	Partial_Sun	L5	DSC	Cement	2	1	47	47
95	95	Shade	L8	DSC	Cement	2	10	47	47
96	96	Chamber	CDC	DSC	Cement	2	0	50	50
97	97	Full_Sun	L3	CTL	Metal	2	0	0	43
98	98	Partial_Sun	L6	CTL	Metal	2	0	0	46
99	99	Shade	L9	CTL	Metal	2	0	0	45

100	100	Chamber	CDC	CTL	Metal	2	0	0	40
101	101	Full_Sun	L3	DLT	Metal	2	25	49	49
102	102	Partial_Sun	L6	DLT	Metal	2	41	44	44
103	103	Shade	L9	DLT	Metal	2	38	46	46
104	104	Chamber	CDC	DLT	Metal	2	44	46	46
105	105	Full_Sun	L3	DSC	Metal	2	40	48	48
106	106	Partial_Sun	L6	DSC	Metal	2	40	51	51
107	107	Shade	L9	DSC	Metal	2	36	45	45
108	108	Chamber	CDC	DSC	Metal	2	25	33	33
109	109	Full_Sun	L1	CTL	Wood	3	0	0	53
110	110	Partial_Sun	L4	CTL	Wood	3	0	0	53
111	111	Shade	L7	CTL	Wood	3	0	0	55
112	112	Chamber	CDC	CTL	Wood	3	0	1	52
113	113	Full_Sun	L1	DLT	Wood	3	0	28	57
114	114	Partial_Sun	L4	DLT	Wood	3	0	5	51
115	115	Shade	L7	DLT	Wood	3	0	2	55
116	116	Chamber	CDC	DLT	Wood	3	0	2	55
117	117	Full_Sun	L1	DSC	Wood	3	0	37	47
118	118	Partial_Sun	L4	DSC	Wood	3	1	43	51
119	119	Shade	L7	DSC	Wood	3	1	40	53
120	120	Chamber	CDC	DSC	Wood	3	0	35	58
121	121	Full_Sun	L2	CTL	Cement	3	0	1	45
122	122	Partial_Sun	L5	CTL	Cement	3	0	0	53
123	123	Shade	L8	CTL	Cement	3	0	0	50
124	124	Chamber	CDC	CTL	Cement	3	0	1	49
125	125	Full_Sun	L2	DLT	Cement	3	3	48	50
126	126	Partial_Sun	L5	DLT	Cement	3	0	40	46
127	127	Shade	L8	DLT	Cement	3	0	34	47
128	128	Chamber	CDC	DLT	Cement	3	0	24	46
129	129	Full_Sun	L2	DSC	Cement	3	2	50	50
130	130	Partial_Sun	L5	DSC	Cement	3	17	52	52
131	131	Shade	L8	DSC	Cement	3	12	51	51
132	132	Chamber	CDC	DSC	Cement	3	3	46	46
133	133	Full_Sun	L3	CTL	Metal	3	0	1	52
134	134	Partial_Sun	L6	CTL	Metal	3	0	2	56
135	135	Shade	L9	CTL	Metal	3	0	2	48
136	136	Chamber	CDC	CTL	Metal	3	0	1	44
137	137	Full_Sun	L3	DLT	Metal	3	15	50	50
138	138	Partial_Sun	L6	DLT	Metal	3	25	51	51
139	139	Shade	L9	DLT	Metal	3	12	49	49
140	140	Chamber	CDC	DLT	Metal	3	51	55	55
141	141	Full_Sun	L3	DSC	Metal	3	54	57	57
142	142	Partial_Sun	L6	DSC	Metal	3	34	52	52
143	143	Shade	L9	DSC	Metal	3	25	50	50
144	144	Chamber	CDC	DSC	Metal	3	53	53	53
145	145	Full_Sun	L1	CTL	Wood	4	0	2	40
146	146	Partial_Sun	L4	CTL	Wood	4	0	1	49
147	147	Shade	L7	CTL	Wood	4	0	0	47
148	148	Chamber	CDC	CTL	Wood	4	0	2	49
149	149	Full_Sun	L1	DLT	Wood	4	1	22	46
150	150	Partial_Sun	L4	DLT	Wood	4	0	11	50
151	151	Shade	L7	DLT	Wood	4	0	14	49
152	152	Chamber	CDC	DLT	Wood	4	2	44	49
153	153	Full_Sun	L1	DSC	Wood	4	0	41	46
154	154	Partial_Sun	L4	DSC	Wood	4	0	28	35
155	155	Shade	L7	DSC	Wood	4	0	46	49
156	156	Chamber	CDC	DSC	Wood	4	3	48	48
157	157	Full_Sun	L2	CTL	Cement	4	0	1	51
158	158	Partial_Sun	L5	CTL	Cement	4	0	3	50
159	159	Shade	L8	CTL	Cement	4	0	2	52
160	160	Chamber	CDC	CTL	Cement	4	0	1	49
161	161	Full_Sun	L2	DLT	Cement	4	0	43	50
162	162	Partial_Sun	L5	DLT	Cement	4	0	34	53
163	163	Shade	L8	DLT	Cement	4	0	33	47
164	164	Chamber	CDC	DLT	Cement	4	0	32	53
165	165	Full_Sun	L2	DSC	Cement	4	1	50	50
166	166	Partial_Sun	L5	DSC	Cement	4	17	53	53
167	167	Shade	L8	DSC	Cement	4	5	47	47
168	168	Chamber	CDC	DSC	Cement	4	15	47	47
169	169	Full_Sun	L3	CTL	Metal	4	0	0	48

170	170	Partial_Sun	L6	CTL	Metal	4	0	0	49
171	171	Shade	L9	CTL	Metal	4	1	1	52
172	172	Chamber	CDC	CTL	Metal	4	0	3	56
173	173	Full_Sun	L3	DLT	Metal	4	26	50	50
174	174	Partial_Sun	L6	DLT	Metal	4	26	44	44
175	175	Shade	L9	DLT	Metal	4	39	51	51
176	176	Chamber	CDC	DLT	Metal	4	49	52	52
177	177	Full_Sun	L3	DSC	Metal	4	27	47	47
178	178	Partial_Sun	L6	DSC	Metal	4	46	51	51
179	179	Shade	L9	DSC	Metal	4	43	50	50
180	180	Chamber	CDC	DSC	Metal	4	51	51	51
181	181	Full_Sun	L1	CTL	Wood	5	0	2	57
182	182	Partial_Sun	L4	CTL	Wood	5	0	1	60
183	183	Shade	L7	CTL	Wood	5	0	2	54
184	184	Chamber	CDC	CTL	Wood	5	0	2	50
185	185	Full_Sun	L1	DLT	Wood	5	0	53	57
186	186	Partial_Sun	L4	DLT	Wood	5	0	31	53
187	187	Shade	L7	DLT	Wood	5	0	24	51
188	188	Chamber	CDC	DLT	Wood	5	0	35	53
189	189	Full_Sun	L1	DSC	Wood	5	0	45	45
190	190	Partial_Sun	L4	DSC	Wood	5	0	54	54
191	191	Shade	L7	DSC	Wood	5	0	47	48
192	192	Chamber	CDC	DSC	Wood	5	0	49	51
193	193	Full_Sun	L2	CTL	Cement	5	0	0	49
194	194	Partial_Sun	L5	CTL	Cement	5	0	0	49
195	195	Shade	L8	CTL	Cement	5	0	3	53
196	196	Chamber	CDC	CTL	Cement	5	0	1	60
197	197	Full_Sun	L2	DLT	Cement	5	0	15	54
198	198	Partial_Sun	L5	DLT	Cement	5	0	12	51
199	199	Shade	L8	DLT	Cement	5	0	20	46
200	200	Chamber	CDC	DLT	Cement	5	0	12	51
201	201	Full_Sun	L2	DSC	Cement	5	0	48	50
202	202	Partial_Sun	L5	DSC	Cement	5	4	52	52
203	203	Shade	L8	DSC	Cement	5	3	50	52
204	204	Chamber	CDC	DSC	Cement	5	4	49	49
205	205	Full_Sun	L3	CTL	Metal	5	0	0	46
206	206	Partial_Sun	L6	CTL	Metal	5	0	0	48
207	207	Shade	L9	CTL	Metal	5	0	1	50
208	208	Chamber	CDC	CTL	Metal	5	0	1	49
209	209	Full_Sun	L3	DLT	Metal	5	26	41	44
210	210	Partial_Sun	L6	DLT	Metal	5	18	45	45
211	211	Shade	L9	DLT	Metal	5	35	50	50
212	212	Chamber	CDC	DLT	Metal	5	46	49	49
213	213	Full_Sun	L3	DSC	Metal	5	40	48	48
214	214	Partial_Sun	L6	DSC	Metal	5	44	48	48
215	215	Shade	L9	DSC	Metal	5	30	48	48
216	216	Chamber	CDC	DSC	Metal	5	49	60	60
217	217	Full_Sun	L1	CTL	Wood	6	0	1	56
218	218	Partial_Sun	L4	CTL	Wood	6	0	3	52
219	219	Shade	L7	CTL	Wood	6	0	2	54
220	220	Chamber	CDC	CTL	Wood	6	0	4	53
221	221	Full_Sun	L1	DLT	Wood	6	0	43	53
222	222	Partial_Sun	L4	DLT	Wood	6	0	12	52
223	223	Shade	L7	DLT	Wood	6	0	18	49
224	224	Chamber	CDC	DLT	Wood	6	0	40	45
225	225	Full_Sun	L1	DSC	Wood	6	0	51	54
226	226	Partial_Sun	L4	DSC	Wood	6	0	44	50
227	227	Shade	L7	DSC	Wood	6	0	38	53
228	228	Chamber	CDC	DSC	Wood	6	13	55	57
229	229	Full_Sun	L2	CTL	Cement	6	0	0	53
230	230	Partial_Sun	L5	CTL	Cement	6	0	5	49
231	231	Shade	L8	CTL	Cement	6	0	8	54
232	232	Chamber	CDC	CTL	Cement	6	0	12	51
233	233	Full_Sun	L2	DLT	Cement	6	0	45	54
234	234	Partial_Sun	L5	DLT	Cement	6	1	41	57
235	235	Shade	L8	DLT	Cement	6	0	19	44
236	236	Chamber	CDC	DLT	Cement	6	0	42	54
237	237	Full_Sun	L2	DSC	Cement	6	0	37	50
238	238	Partial_Sun	L5	DSC	Cement	6	12	50	50
239	239	Shade	L8	DSC	Cement	6	21	51	51

240	240	Chamber	CDC	DSC	Cement	6	36	59	59
241	241	Full_Sun	L3	CTL	Metal	6	0	1	53
242	242	Partial_Sun	L6	CTL	Metal	6	0	0	58
243	243	Shade	L9	CTL	Metal	6	0	1	56
244	244	Chamber	CDC	CTL	Metal	6	0	2	57
245	245	Full_Sun	L3	DLT	Metal	6	16	49	57
246	246	Partial_Sun	L6	DLT	Metal	6	34	54	54
247	247	Shade	L9	DLT	Metal	6	23	52	55
248	248	Chamber	CDC	DLT	Metal	6	48	48	48
249	249	Full_Sun	L3	DSC	Metal	6	29	56	56
250	250	Partial_Sun	L6	DSC	Metal	6	47	50	50
251	251	Shade	L9	DSC	Metal	6	37	52	52
252	252	Chamber	CDC	DSC	Metal	6	40	47	47
253	253	Full_Sun	L1	CTL	Wood	7	0	1	50
254	254	Partial_Sun	L4	CTL	Wood	7	0	3	48
255	255	Shade	L7	CTL	Wood	7	1	2	50
256	256	Chamber	CDC	CTL	Wood	7	0	0	49
257	257	Full_Sun	L1	DLT	Wood	7	1	25	39
258	258	Partial_Sun	L4	DLT	Wood	7	0	19	39
259	259	Shade	L7	DLT	Wood	7	0	11	60
260	260	Chamber	CDC	DLT	Wood	7	0	19	54
261	261	Full_Sun	L1	DSC	Wood	7	0	40	47
262	262	Partial_Sun	L4	DSC	Wood	7	0	23	47
263	263	Shade	L7	DSC	Wood	7	0	17	42
264	264	Chamber	CDC	DSC	Wood	7	0	17	53
265	265	Full_Sun	L2	CTL	Cement	7	0	0	54
266	266	Partial_Sun	L5	CTL	Cement	7	0	2	46
267	267	Shade	L8	CTL	Cement	7	0	0	47
268	268	Chamber	CDC	CTL	Cement	7	0	3	42
269	269	Full_Sun	L2	DLT	Cement	7	0	6	45
270	270	Partial_Sun	L5	DLT	Cement	7	0	13	51
271	271	Shade	L8	DLT	Cement	7	0	5	47
272	272	Chamber	CDC	DLT	Cement	7	0	9	45
273	273	Full_Sun	L2	DSC	Cement	7	4	46	46
274	274	Partial_Sun	L5	DSC	Cement	7	0	45	47
275	275	Shade	L8	DSC	Cement	7	0	44	48
276	276	Chamber	CDC	DSC	Cement	7	6	45	45
277	277	Full_Sun	L3	CTL	Metal	7	0	0	50
278	278	Partial_Sun	L6	CTL	Metal	7	0	0	54
279	279	Shade	L9	CTL	Metal	7	0	1	54
280	280	Chamber	CDC	CTL	Metal	7	0	0	46
281	281	Full_Sun	L3	DLT	Metal	7	3	31	46
282	282	Partial_Sun	L6	DLT	Metal	7	11	41	45
283	283	Shade	L9	DLT	Metal	7	2	43	52
284	284	Chamber	CDC	DLT	Metal	7	53	59	59
285	285	Full_Sun	L3	DSC	Metal	7	36	54	56
286	286	Partial_Sun	L6	DSC	Metal	7	32	56	58
287	287	Shade	L9	DSC	Metal	7	16	39	40
288	288	Chamber	CDC	DSC	Metal	7	45	53	53
289	289	Full_Sun	L1	CTL	Wood	8	0	0	51
290	290	Partial_Sun	L4	CTL	Wood	8	0	0	59
291	291	Shade	L7	CTL	Wood	8	0	1	49
292	292	Chamber	CDC	CTL	Wood	8	0	0	45
293	293	Full_Sun	L1	DLT	Wood	8	0	2	44
294	294	Partial_Sun	L4	DLT	Wood	8	0	4	43
295	295	Shade	L7	DLT	Wood	8	0	1	43
296	296	Chamber	CDC	DLT	Wood	8	0	14	51
297	297	Full_Sun	L1	DSC	Wood	8	0	19	49
298	298	Partial_Sun	L4	DSC	Wood	8	0	20	52
299	299	Shade	L7	DSC	Wood	8	0	12	56
300	300	Chamber	CDC	DSC	Wood	8	0	22	53
301	301	Full_Sun	L2	CTL	Cement	8	0	1	51
302	302	Partial_Sun	L5	CTL	Cement	8	0	1	49
303	303	Shade	L8	CTL	Cement	8	0	0	45
304	304	Chamber	CDC	CTL	Cement	8	0	1	51
305	305	Full_Sun	L2	DLT	Cement	8	0	39	50
306	306	Partial_Sun	L5	DLT	Cement	8	0	19	58
307	307	Shade	L8	DLT	Cement	8	0	11	50
308	308	Chamber	CDC	DLT	Cement	8	0	6	51
309	309	Full_Sun	L2	DSC	Cement	8	0	45	46

310	310	Partial_Sun	L5	DSC	Cement	8	0	47	47
311	311	Shade	L8	DSC	Cement	8	3	44	45
312	312	Chamber	CDC	DSC	Cement	8	0	42	42
313	313	Full_Sun	L3	CTL	Metal	8	0	0	52
314	314	Partial_Sun	L6	CTL	Metal	8	0	1	45
315	315	Shade	L9	CTL	Metal	8	0	2	46
316	316	Chamber	CDC	CTL	Metal	8	0	0	52
317	317	Full_Sun	L3	DLT	Metal	8	9	38	49
318	318	Partial_Sun	L6	DLT	Metal	8	5	15	52
319	319	Shade	L9	DLT	Metal	8	8	31	47
320	320	Chamber	CDC	DLT	Metal	8	49	52	52
321	321	Full_Sun	L3	DSC	Metal	8	15	45	47
322	322	Partial_Sun	L6	DSC	Metal	8	42	51	51
323	323	Shade	L9	DSC	Metal	8	21	51	51
324	324	Chamber	CDC	DSC	Metal	8	47	54	54
325	325	Full_Sun	L1	CTL	Wood	9	0	0	53
326	326	Partial_Sun	L4	CTL	Wood	9	0	1	48
327	327	Shade	L7	CTL	Wood	9	0	0	51
328	328	Chamber	CDC	CTL	Wood	9	0	1	57
329	329	Full_Sun	L1	DLT	Wood	9	0	7	50
330	330	Partial_Sun	L4	DLT	Wood	9	0	4	46
331	331	Shade	L7	DLT	Wood	9	0	1	49
332	332	Chamber	CDC	DLT	Wood	9	0	22	51
333	333	Full_Sun	L1	DSC	Wood	9	2	43	51
334	334	Partial_Sun	L4	DSC	Wood	9	1	43	55
335	335	Shade	L7	DSC	Wood	9	0	34	51
336	336	Chamber	CDC	DSC	Wood	9	2	44	49
337	337	Full_Sun	L2	CTL	Cement	9	0	0	39
338	338	Partial_Sun	L5	CTL	Cement	9	0	0	57
339	339	Shade	L8	CTL	Cement	9	0	1	51
340	340	Chamber	CDC	CTL	Cement	9	0	0	50
341	341	Full_Sun	L2	DLT	Cement	9	0	45	47
342	342	Partial_Sun	L5	DLT	Cement	9	0	8	47
343	343	Shade	L8	DLT	Cement	9	0	3	43
344	344	Chamber	CDC	DLT	Cement	9	0	29	47
345	345	Full_Sun	L2	DSC	Cement	9	2	53	53
346	346	Partial_Sun	L5	DSC	Cement	9	3	50	50
347	347	Shade	L8	DSC	Cement	9	15	46	46
348	348	Chamber	CDC	DSC	Cement	9	20	54	54
349	349	Full_Sun	L3	CTL	Metal	9	0	2	51
350	350	Partial_Sun	L6	CTL	Metal	9	0	0	48
351	351	Shade	L9	CTL	Metal	9	0	0	46
352	352	Chamber	CDC	CTL	Metal	9	1	0	46
353	353	Full_Sun	L3	DLT	Metal	9	14	37	50
354	354	Partial_Sun	L6	DLT	Metal	9	9	40	41
355	355	Shade	L9	DLT	Metal	9	6	34	49
356	356	Chamber	CDC	DLT	Metal	9	38	45	45
357	357	Full_Sun	L3	DSC	Metal	9	46	50	50
358	358	Partial_Sun	L6	DSC	Metal	9	46	55	55
359	359	Shade	L9	DSC	Metal	9	45	56	56
360	360	Chamber	CDC	DSC	Metal	9	54	58	58
361	361	Full_Sun	L1	CTL	Wood	12	0	0	50
362	362	Partial_Sun	L4	CTL	Wood	12	0	0	50
363	363	Shade	L7	CTL	Wood	12	0	0	47
364	364	Chamber	CDC	CTL	Wood	12	0	0	52
365	365	Full_Sun	L1	DLT	Wood	12	0	4	52
366	366	Partial_Sun	L4	DLT	Wood	12	0	0	51
367	367	Shade	L7	DLT	Wood	12	0	0	54
368	368	Chamber	CDC	DLT	Wood	12	0	14	52
369	369	Full_Sun	L1	DSC	Wood	12	0	22	53
370	370	Partial_Sun	L4	DSC	Wood	12	0	20	52
371	371	Shade	L7	DSC	Wood	12	0	16	53
372	372	Chamber	CDC	DSC	Wood	12	0	42	57
373	373	Full_Sun	L2	CTL	Cement	12	0	0	53
374	374	Partial_Sun	L5	CTL	Cement	12	0	0	51
375	375	Shade	L8	CTL	Cement	12	0	0	51
376	376	Chamber	CDC	CTL	Cement	12	0	0	47
377	377	Full_Sun	L2	DLT	Cement	12	0	21	57
378	378	Partial_Sun	L5	DLT	Cement	12	0	4	46
379	379	Shade	L8	DLT	Cement	12	0	3	49

```
380 380     Chamber  CDC       DLT  Cement   12    0     16    52
381 381    Full_Sun   L2       DSC  Cement   12    2     51    51
382 382 Partial_Sun   L5       DSC  Cement   12    0     48    48
383 383       Shade   L8       DSC  Cement   12    5     51    51
384 384     Chamber  CDC       DSC  Cement   12    4     55    55
385 385    Full_Sun   L3       CTL   Metal   12    0      0    40
386 386 Partial_Sun   L6       CTL   Metal   12    0      0    45
387 387       Shade   L9       CTL   Metal   12    0      0    46
388 388     Chamber  CDC       CTL   Metal   12    0      0    44
389 389    Full_Sun   L3       DLT   Metal   12    0     33    56
390 390 Partial_Sun   L6       DLT   Metal   12   10     56    59
391 391       Shade   L9       DLT   Metal   12    0     25    53
392 392     Chamber  CDC       DLT   Metal   12   41     50    50
393 393    Full_Sun   L3       DSC   Metal   12   38     44    44
394 394 Partial_Sun   L6       DSC   Metal   12   32     43    44
395 395       Shade   L9       DSC   Metal   12   20     45    45
396 396     Chamber  CDC       DSC   Metal   12   48     48    48
> model_all <- glm(cbind(KD30,Total-KD30)~Exposure+Treatment+Surface+Time+Treatment*Surface, fami
ly="binomial", data=mydata)
> summary(model_all)

Call:
glm(formula = cbind(KD30, Total - KD30) ~ Exposure + Treatment + 
    Surface + Time + Treatment * Surface, family = "binomial", 
    data = mydata)

Deviance Residuals: 
    Min       1Q   Median       3Q      Max  
-6.0147  -0.8856  -0.1568  -0.0001   8.1226  

Coefficients:
                             Estimate Std. Error z value Pr(>|z|)    
(Intercept)                 -17.95069  435.94251  -0.041    0.967    
ExposureFull_Sun             -1.54389    0.08863 -17.419   <2e-16 ***
ExposurePartial_Sun          -1.16738    0.08760 -13.327   <2e-16 ***
ExposureShade                -1.53718    0.08968 -17.140   <2e-16 ***
TreatmentDLT                 18.83614  435.94252   0.043    0.966    
TreatmentDSC                 20.37550  435.94252   0.047    0.963    
SurfaceMetal                 13.98741  435.94309   0.032    0.974    
SurfaceWood                  13.06895  435.94366   0.030    0.976    
Time1                        -2.55672    0.14793 -17.283   <2e-16 ***
Time2                        -3.25522    0.15560 -20.920   <2e-16 ***
Time3                        -3.66052    0.15533 -23.567   <2e-16 ***
Time4                        -3.06409    0.15298 -20.029   <2e-16 ***
Time5                        -3.61268    0.15626 -23.119   <2e-16 ***
Time6                        -3.19268    0.15097 -21.148   <2e-16 ***
Time7                        -4.59856    0.16188 -28.407   <2e-16 ***
Time8                        -4.66274    0.16271 -28.656   <2e-16 ***
Time9                        -3.64626    0.15580 -23.403   <2e-16 ***
Time12                       -4.56644    0.16124 -28.321   <2e-16 ***
TreatmentDLT:SurfaceMetal    -9.91510  435.94311  -0.023    0.982    
TreatmentDSC:SurfaceMetal   -10.23381  435.94310  -0.023    0.981    
TreatmentDLT:SurfaceWood    -17.21598  435.94391  -0.039    0.968    
TreatmentDSC:SurfaceWood    -17.15896  435.94372  -0.039    0.969    
---
Signif. codes:  0 ‘***’ 0.001 ‘**’ 0.01 ‘*’ 0.05 ‘.’ 0.1 ‘ ’ 1

(Dispersion parameter for binomial family taken to be 1)

    Null deviance: 13645.6  on 395  degrees of freedom
Residual deviance:  1567.8  on 374  degrees of freedom
AIC: 2082.3

Number of Fisher Scoring iterations: 17

> #step down by removing non-sig factors
> model_sd1 <- glm(cbind(KD30,Total-KD30)~Exposure+Time, family="binomial", data=mydata)
> summary(model_sd1)

Call:
```

```
glm(formula = cbind(KD30, Total - KD30) ~ Exposure + Time, family = "binomial",
    data = mydata)

Deviance Residuals:
    Min       1Q   Median       3Q      Max
-8.4668  -4.3526  -3.6733   0.3166  13.6238

Coefficients:
                    Estimate Std. Error z value Pr(>|z|)
(Intercept)         -0.22357    0.05527  -4.045 5.23e-05 ***
ExposureFull_Sun    -0.52091    0.05174 -10.068  < 2e-16 ***
ExposurePartial_Sun -0.41766    0.05047  -8.276  < 2e-16 ***
ExposureShade       -0.54975    0.05194 -10.585  < 2e-16 ***
Time1               -0.63887    0.07290  -8.764  < 2e-16 ***
Time2               -0.93789    0.07863 -11.928  < 2e-16 ***
Time3               -1.02577    0.07794 -13.162  < 2e-16 ***
Time4               -0.80924    0.07569 -10.692  < 2e-16 ***
Time5               -1.06109    0.07857 -13.504  < 2e-16 ***
Time6               -0.88039    0.07500 -11.739  < 2e-16 ***
Time7               -1.42061    0.08712 -16.306  < 2e-16 ***
Time8               -1.49792    0.08854 -16.918  < 2e-16 ***
Time9               -1.01041    0.07836 -12.894  < 2e-16 ***
Time12              -1.50962    0.08833 -17.090  < 2e-16 ***
---
Signif. codes:  0 ‘***’ 0.001 ‘**’ 0.01 ‘*’ 0.05 ‘.’ 0.1 ‘ ’ 1

(Dispersion parameter for binomial family taken to be 1)

    Null deviance: 13646  on 395  degrees of freedom
Residual deviance: 12925  on 382  degrees of freedom
AIC: 13423

Number of Fisher Scoring iterations: 5

> #do pairwise comparison for exposure using multcomp library
> library(multcomp)
Loading required package: mvtnorm
Loading required package: survival
Loading required package: TH.data
Loading required package: MASS

Attaching package: ‘TH.data’

The following object is masked from ‘package:MASS’:

    geyser

> Tukey_exposure <-glht(model_sd1, linfct=mcp(Exposure="Tukey"))
> summary(Tukey_exposure)

         Simultaneous Tests for General Linear Hypotheses

Multiple Comparisons of Means: Tukey Contrasts


Fit: glm(formula = cbind(KD30, Total - KD30) ~ Exposure + Time, family = "binomial",
    data = mydata)

Linear Hypotheses:
                            Estimate Std. Error z value Pr(>|z|)
Full_Sun - Chamber == 0     -0.52091    0.05174 -10.068   <0.001 ***
Partial_Sun - Chamber == 0  -0.41766    0.05047  -8.276   <0.001 ***
Shade - Chamber == 0        -0.54975    0.05194 -10.585   <0.001 ***
Partial_Sun - Full_Sun == 0  0.10326    0.05420   1.905   0.2255
Shade - Full_Sun == 0       -0.02884    0.05557  -0.519   0.9545
Shade - Partial_Sun == 0    -0.13210    0.05436  -2.430   0.0717 .
---
Signif. codes:  0 ‘***’ 0.001 ‘**’ 0.01 ‘*’ 0.05 ‘.’ 0.1 ‘ ’ 1
(Adjusted p values reported -- single-step method)
```

```
> Tukey_time <-glht(model_sd1, linfct=mcp(Time="Tukey"))
> summary(Tukey_Time)
Error in summary(Tukey_Time) : object 'Tukey_Time' not found
> summary(Tukey_time)

         Simultaneous Tests for General Linear Hypotheses

Multiple Comparisons of Means: Tukey Contrasts


Fit: glm(formula = cbind(KD30, Total - KD30) ~ Exposure + Time, family = "binomial",
    data = mydata)

Linear Hypotheses:
               Estimate Std. Error z value Pr(>|z|)
1 - 0.25 == 0  -0.63887    0.07290  -8.764  < 0.001 ***
2 - 0.25 == 0  -0.93789    0.07863 -11.928  < 0.001 ***
3 - 0.25 == 0  -1.02577    0.07794 -13.162  < 0.001 ***
4 - 0.25 == 0  -0.80924    0.07569 -10.692  < 0.001 ***
5 - 0.25 == 0  -1.06109    0.07857 -13.504  < 0.001 ***
6 - 0.25 == 0  -0.88039    0.07500 -11.739  < 0.001 ***
7 - 0.25 == 0  -1.42061    0.08712 -16.306  < 0.001 ***
8 - 0.25 == 0  -1.49792    0.08854 -16.918  < 0.001 ***
9 - 0.25 == 0  -1.01041    0.07836 -12.894  < 0.001 ***
12 - 0.25 == 0 -1.50962    0.08833 -17.090  < 0.001 ***
2 - 1 == 0     -0.29902    0.08491  -3.521  0.01817 *
3 - 1 == 0     -0.38691    0.08426  -4.592  < 0.001 ***
4 - 1 == 0     -0.17038    0.08219  -2.073  0.59152
5 - 1 == 0     -0.42222    0.08484  -4.976  < 0.001 ***
6 - 1 == 0     -0.24152    0.08155  -2.962  0.10211
7 - 1 == 0     -0.78174    0.09281  -8.423  < 0.001 ***
8 - 1 == 0     -0.85905    0.09414  -9.125  < 0.001 ***
9 - 1 == 0     -0.37155    0.08465  -4.389  < 0.001 ***
12 - 1 == 0    -0.87075    0.09395  -9.269  < 0.001 ***
3 - 2 == 0     -0.08789    0.08926  -0.985  0.99620
4 - 2 == 0      0.12864    0.08731   1.473  0.92700
5 - 2 == 0     -0.12320    0.08981  -1.372  0.95411
6 - 2 == 0      0.05750    0.08671   0.663  0.99988
7 - 2 == 0     -0.48272    0.09737  -4.957  < 0.001 ***
8 - 2 == 0     -0.56003    0.09864  -5.677  < 0.001 ***
9 - 2 == 0     -0.07253    0.08963  -0.809  0.99928
12 - 2 == 0    -0.57173    0.09846  -5.807  < 0.001 ***
4 - 3 == 0      0.21653    0.08667   2.498  0.30087
5 - 3 == 0     -0.03531    0.08919  -0.396  1.00000
6 - 3 == 0      0.14539    0.08607   1.689  0.83836
7 - 3 == 0     -0.39483    0.09680  -4.079  0.00240 **
8 - 3 == 0     -0.47214    0.09808  -4.814  < 0.001 ***
9 - 3 == 0      0.01536    0.08901   0.173  1.00000
12 - 3 == 0    -0.48384    0.09789  -4.943  < 0.001 ***
5 - 4 == 0     -0.25184    0.08723  -2.887  0.12490
6 - 4 == 0     -0.07114    0.08404  -0.847  0.99893
7 - 4 == 0     -0.61136    0.09500  -6.436  < 0.001 ***
8 - 4 == 0     -0.68867    0.09630  -7.151  < 0.001 ***
9 - 4 == 0     -0.20117    0.08704  -2.311  0.42096
12 - 4 == 0    -0.70037    0.09611  -7.288  < 0.001 ***
6 - 5 == 0      0.18070    0.08664   2.086  0.58233
7 - 5 == 0     -0.35952    0.09730  -3.695  0.00969 **
8 - 5 == 0     -0.43683    0.09857  -4.432  < 0.001 ***
9 - 5 == 0      0.05067    0.08955   0.566  0.99997
12 - 5 == 0    -0.44853    0.09838  -4.559  < 0.001 ***
7 - 6 == 0     -0.54022    0.09445  -5.719  < 0.001 ***
8 - 6 == 0     -0.61753    0.09576  -6.449  < 0.001 ***
9 - 6 == 0     -0.13003    0.08645  -1.504  0.91714
12 - 6 == 0    -0.62923    0.09557  -6.584  < 0.001 ***
8 - 7 == 0     -0.07731    0.10551  -0.733  0.99970
9 - 7 == 0      0.41019    0.09713   4.223  0.00120 **
12 - 7 == 0    -0.08901    0.10533  -0.845  0.99895
9 - 8 == 0      0.48750    0.09841   4.954  < 0.001 ***
12 - 8 == 0    -0.01170    0.10650  -0.110  1.00000
12 - 9 == 0    -0.49920    0.09822  -5.083  < 0.001 ***
```

```
---
Signif. codes:  0 ‘***’ 0.001 ‘**’ 0.01 ‘*’ 0.05 ‘.’ 0.1 ‘ ’ 1
(Adjusted p values reported -- single-step method)

Warning messages:
1: In RET$pfunction("adjusted", ...) : Completion with error > abseps
2: In RET$pfunction("adjusted", ...) : Completion with error > abseps
3: In RET$pfunction("adjusted", ...) : Completion with error > abseps
4: In RET$pfunction("adjusted", ...) : Completion with error > abseps
5: In RET$pfunction("adjusted", ...) : Completion with error > abseps
>
```
